# Bone morphogenetic protein and Notch signalling crosstalk in poor‐prognosis, mesenchymal‐subtype colorectal cancer

**DOI:** 10.1002/path.4891

**Published:** 2017-05-03

**Authors:** Shazia Irshad, Mukesh Bansal, Paolo Guarnieri, Hayley Davis, Ayman Al Haj Zen, Brygida Baran, Claudia Maria Assunta Pinna, Haseeb Rahman, Sujata Biswas, Chiara Bardella, Rosemary Jeffery, Lai Mun Wang, James Edward East, Ian Tomlinson, Annabelle Lewis, Simon John Leedham

**Affiliations:** ^1^ Gastrointestinal Stem‐cell Biology Laboratory, Oxford Centre for Cancer Gene Research, Wellcome Trust Centre for Human GeneticsUniversity of OxfordOxfordUK; ^2^ Department of Systems BiologyColumbia University Medical CenterNew YorkNYUSA; ^3^ PsychoGenics Inc., 765 Old Saw Mill River RoadTarrytownNYUSA; ^4^ Wellcome Trust Centre For Human Genetics, Division of Cardiovascular Medicine, Radcliffe Department of MedicineUniversity of OxfordOxfordUK; ^5^ Department of Genetics, Faculty of Biology and Environmental ProtectionUniversity of SilesiaKatowicePoland; ^6^ Department of Surgery, Oncology and GastroenterologyUniversity Hospital PadovaPadovaItaly; ^7^ Department of Biological and Medical SciencesOxford Brookes UniversityOxfordUK; ^8^ Molecular and Population Genetics Laboratory, Oxford Centre for Cancer Gene Research, Wellcome Trust Centre for Human GeneticsUniversity of OxfordOxfordUK; ^9^ Colorectal Cancer Genetics, Centre for Digestive Diseases, Blizard Institute, Barts and the London School of Medicine and DentistryLondonUK; ^10^ Cellular Pathology, Level 1John Radcliffe HospitalOxfordUK; ^11^ Translational Gastroenterology Unit, Experimental Medicine Division, Nuffield Department of Clinical MedicineJohn Radcliffe HospitalOxfordUK

**Keywords:** BMP, Notch, EMT, colorectal cancer subtypes

## Abstract

The functional role of bone morphogenetic protein (BMP) signalling in colorectal cancer (CRC) is poorly defined, with contradictory results in cancer cell line models reflecting the inherent difficulties of assessing a signalling pathway that is context‐dependent and subject to genetic constraints. By assessing the transcriptional response of a diploid human colonic epithelial cell line to BMP ligand stimulation, we generated a prognostic BMP signalling signature, which was applied to multiple CRC datasets to investigate BMP heterogeneity across CRC molecular subtypes. We linked BMP and Notch signalling pathway activity and function in human colonic epithelial cells, and normal and neoplastic tissue. BMP induced Notch through a γ‐secretase‐independent interaction, regulated by the SMAD proteins. In homeostasis, BMP/Notch co‐localization was restricted to cells at the top of the intestinal crypt, with more widespread interaction in some human CRC samples. BMP signalling was downregulated in the majority of CRCs, but was conserved specifically in mesenchymal‐subtype tumours, where it interacts with Notch to induce an epithelial–mesenchymal transition (EMT) phenotype. In intestinal homeostasis, BMP–Notch pathway crosstalk is restricted to differentiating cells through stringent pathway segregation. Conserved BMP activity and loss of signalling stringency in mesenchymal‐subtype tumours promotes a synergistic BMP–Notch interaction, and this correlates with poor patient prognosis. BMP signalling heterogeneity across CRC subtypes and cell lines can account for previous experimental contradictions. Crosstalk between the BMP and Notch pathways will render mesenchymal‐subtype CRC insensitive to γ‐secretase inhibition unless BMP activation is concomitantly addressed. © 2017 The Authors. Journal of Pathology published by John Wiley & Sons Ltd on behalf of Pathological Society of Great Britain and Ireland.

## Introduction

In adult tissue homeostasis, stringent control of stem cell division and daughter cell fate determination is achieved by interactions of various signalling pathways. These signalling cascades consist of secreted ligands (morphogens), receptors and antagonists, with overlapping expression of pathway constituents allowing synergistic or antagonistic interactions. It is the complexity of these intracellular networks, amplifying or attenuating the biological effects of individual pathways in a context‐dependent fashion, that generates a powerful mechanism for conferring diverse cellular functions on various tissues 1. The intestinal epithelium, with its stereotypical architecture, is a paradigm for morphogenic cell fate control, as it undergoes continual replacement supported by intestinal stem cells at the base of the intestinal crypt. Daughter cells, generated by asymmetrical stem cell division, pass along the vertical axis of the intestine, proliferate in the transit‐amplifying zone, and then mature and undergo terminal differentiation as they approach the luminal surface. The epithelial cell response to morphogens is determined by position along the crypt–villus axis as cells migrate through intercompartmental Wnt, Hedgehog, bone morphogenetic protein (BMP) and Notch morphogen gradients 2.

BMPs are members of the transforming growth factor (TGF)‐β superfamily. Secreted BMP ligands bind and activate cell surface BMPR1A (ALK3), BMPR1B (ALK6) and BMPRII receptors, with signal transduction being achieved by C‐terminal phosphorylation of the Mothers against decapentaplegic, Drosophila (SMAD) 1, 5 and 8 proteins. Activated p‐SMAD1/5/8 complexes with SMAD4, translocates to the nucleus, and induces expression of target genes such as Inhibitor of DNA binding (ID) to initiate anti‐proliferative and pro‐differentiation transcription programmes 2. The four mammalian Notch genes encode transmembrane receptors that are activated by neighbouring cell surface ligands from the Jagged and Delta‐like families. Ligand–receptor binding renders the intracellular subunit of the receptor susceptible to proteolytic cleavage by γ‐secretase, releasing the Notch intracellular domain (NICD) into the cytosol. Translocation of cleaved NICD in the nucleus derepresses and activates the expression of Hairy and enhancer of Split1 related (HES and HEY) target genes by binding to the transcription factor CSL and the co‐activator Mastermind‐like 1. Lateral inhibition between Notch‐activated and neighbouring cells mediates binary cell fate decisions, and, in the intestine, this pathway is important for regulating secretory versus enterocyte differentiation programmes in the upper part of the crypt. Murine models have also shown that crypt base Notch signalling promotes progenitor cell proliferation, implicating Notch as a key pleiotropic signalling pathway with a variable effect on intestinal cell function, depending on cell position 3.

Consistent with its role in intestinal homeostasis, dysregulation of BMP signalling can promote intestinal tumourigenesis, with highly penetrant germline mutations in the BMP pathway being responsible for juvenile polyposis syndrome 4, 5 and hereditary mixed polyposis syndrome 6.

Furthermore, genetic variation in the BMP pathway is an important determinant of sporadic colorectal cancer (CRC) risk, with predisposing single‐nucleotide polymorphisms having identified at ligand, antagonist and intracellular effector levels of the pathway 7. Despite this, the precise role of BMP signalling in intestinal neoplasia is poorly defined. Some studies have demonstrated increased expression of BMP ligands as lesions progress through the adenoma–carcinoma sequence 8, 9, whereas others have reported inactivation of the pathway at the cancer transition stage 10, 11. In vitro results from cancer cell lines are contrasting, with BMP signalling inducing cytostasis and differentiation 12, 13 or, alternatively, promoting cell motility, invasiveness 8, 14, epithelial–mesenchymal transition (EMT) 15, 16 and increased tumour volume 17 in different experimental contexts. Subsequent work has shown considerable genetic constraints on pathway activity, with variable effects of BMP signalling according to the underlying SMAD4 and p53 mutation status of the cancer cell lines used 18, 19. Cumulatively, this work highlights the inherent difficulties in assessing signalling pathway activity in cancer cells. The functional effects of BMP signalling are dependent on endogenous pathway constituent expression, the activity of interacting morphogens, genetic constraints, and cellular context, all of which may differ in cancer cell populations.

Recent molecular stratification and analysis by the Colorectal Cancer Subtyping Consortium has revealed considerable disease heterogeneity, and defined four distinct CRC subtypes [consensus molecular subtypes (CMSs) 1–4], each with a unique pathogenic molecular pathway, and with variable responses to treatment and prognosis. CMS1 [microsatellite instability (MSI)] is characterized by hypermutation, MSI, and strong immune system activation (14% of cases). CMS2 (canonical) is driven by Wnt pathway activation, classically through adenomatous polyposis coli mutation (37%). CMS3 (metabolic) is characterized by metabolic dysregulation (13%). CMS4 (mesenchymal) tumours have TGF‐β activation and stromal invasion, and carry the worst prognosis of all of the subtypes (23%). A further 13% of cases are indeterminate 20.

As the functional effect of the BMP pathway is contextually dependent, we wished to investigate whether the activity of this critical homeostatic pathway varies according to CRC molecular subtype. To investigate this, we assessed the transcriptional response of an immortalized human colonic epithelial cell (HCEC) line, derived from a normal crypt explant 21, to recombinant BMP ligand stimulation. From this, we generated a BMP gene signature, which we assessed in multiple CRC datasets. Here, we show that BMP signalling interacts with intracellular Notch signalling to promote an EMT phenotype in intestinal epithelial cells, and that this interaction is relevant in clinically aggressive CRC subtypes.

## Materials and methods

### Ethics

Ethical approval for the use of archival tissue was provided by the South‐West Hampshire Research Ethics Committee A (REC 06/Q1702/99), and that for for endoscopic samples was provided by Oxfordshire Research Ethics Committee A (REC 10/H0604/72). Informed consent was obtained from all patients.

### Cell culture and analysis

HCECs were cultured as described by Roig et al 21. HCECs were subjected to treatment with recombinant proteins and signalling pathway inhibitors, and small interfering RNA (siRNA)‐mediated gene knockdown, and were used in chromatin immunoprecipitation (ChIP) 22 and cell migration experiments as described in supplementary material, Supplementary materials and methods. The primers used in ChIP experiments are listed in supplementary material, Table S1.

### Human tissue sections

Formalin‐fixed, paraffin‐embedded sections (4 µm) were de‐waxed in xylene and rehydrated through graded alcohols to water. De‐waxed sections were subjected to immunohistochemical and in situ analysis 23 as described in supplementary material, Supplementary materials and methods.

### Immunofluorescence

Cultured cells were protected from the light, fixed, washed, and incubated with primary antibodies, as described in supplementary material, Supplementary materials and methods.

### Isolation of individual human colonic crypts

Colonic biopsies from three different patients were washed with phosphate‐buffered saline and incubated in 5 ml of dissociation medium [30 mm EDTA in Dulbecco's modified Eagle's medium without Ca^2+^ and Mg^2+^, 0.5 mm dithiothreitol, and 2% RNAlater (Life Technologies)] for 10 min at room temperature. Tissue was shaken vigorously for 30 s to release individual crypts. Ten single crypts from each patient were hemisected with fine needles under a dissecting microscope, and individual hemicrypts were then aspirated. RNA was extracted, preamplified with the TaqMan PreAmp kit (Applied Biosystems), and reverse transcribed prior to quantitative polymerase chain reaction (PCR).

### Gene expression arrays

For gene expression profiling, we used three controls and three experimental groups for each time point (4 and 24 h). Samples were profiled by the use of Illumina gene expression arrays (Human‐HT‐12v4 expression BeadChip). Details of data processing are described in supplementary material, Supplementary materials and methods.

### Gene set enrichment analysis (GSEA)

GSEA was performed as described by Lin et al 24 and in supplementary material, Supplementary materials and methods. A list of genes used in the enrichment analysis is given in supplementary material, Table 6B1 25, 26, 27, 28.

### Kaplan–Meier survival analysis

K‐means clustering was used to identify two patient groups with similar gene expression levels by use of the ‘k‐means’ function from the Statistics Toolbox in MATLAB. Kaplan–Meier survival curves were generated by use of a MATLAB script, and p‐values were computed with a log‐rank test.

### Human CRC patient cohorts

RNA‐sequencing data from sporadic CRC patients was downloaded from The Cancer Genome Atlas (TCGA) data portal (https://cancergenome.nih.gov) and RSEM‐normalized as distributed by TCGA. Tumour samples were matched to published CRC subtypes 20, 29, 30. If, for any given patient, multiple samples were profiled, we randomly selected one sample for further analysis. anova was used to compare the mean expression values of BMP4, BMP2, BMPR1A, BMPR1B, BMPRII, SMAD1, SMAD4 and SMAD5 across subtypes.

## Results

### Global transcriptional profiling of BMP2/4‐treated HCECs reveals overlapping target gene expression

**Figure 1:**
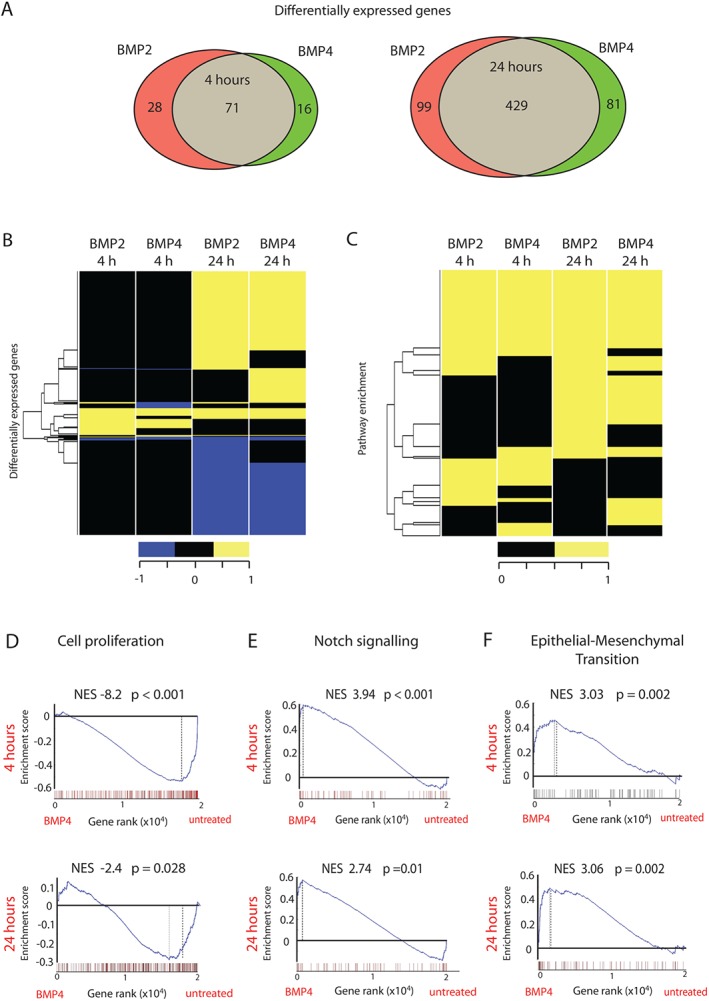
Global transcriptional profiling of BMP2/4‐treated HCECs. (A) Venn diagram showing overlapping and uniquely DEGs (fold change of >2, FDR of <0.05) between BMP2‐treated or BMP4‐treated as compared with untreated HCECs at 4 h or 24 h post‐stimulation. (B) Heat map visualization of changes in genes significantly regulated by at least one treatment (BMP2 or BMP4) after 4 or 24 h. (C) Heat map showing upregulated pathways from the MSigDB‐C2 database (Broad Institute) that were significant in at least one condition following BMP2 or BMP4 treatment of HCECs. (D–F) GSEA in BMP4‐treated versus untreated HCECs using a priori gene sets for cell proliferation (D), Notch signalling (E) and EMT (F) signatures (supplementary material, Table S6B1–S6B4).

Previous studies have shown variable expression levels of BMP2 and BMP4 in CRC 8. In order to determine the downstream effectors of these ligands in intestinal epithelium, we treated diploid, immortalized HCECs with recombinant human BMP2 or BMP4, and assessed global gene expression changes by microarray analysis at 4 and 24 h post‐treatment, to capture both early and secondary changes in expression (supplementary material, Table S2). Using a fold‐change of >2 and a false discovery rate (FDR) of <0.05, we identified 99 (67 upregulated and 32 downregulated) differentially expressed genes (DEGs) induced by BMP2 and 87 (51 upregulated and 36 downregulated) DEGs whose differential expression was caused by BMP4 at 4 h post‐treatment (supplementary material, Tables S3–S5). Using the same cut‐off criteria, we identified 528 (279 upregulated and 249 downregulated) DEGs induced by BMP2 and 510 (281 upregulated and 229 downregulated) DEGs whose differential expression was caused by BMP4 at 24 h post‐treatment as compared with vehicle‐only treated cells (Figure 1A; supplementary material, Tables S3–S5). We observed 82% and 84% overlap between BMP2‐induced and BMP4‐induced target genes at 4 and 24 h respectively, implying functional redundancy between these ligands (Figure 1B; supplementary material, Table S5). Extending our search to identify biological processes affected by BMP2 or BMP4, we computed enrichment scores 31 for curated gene sets available in the C2 database 32, and found a statistically significant overlap in the number of downstream pathways affected by these two effectors of BMP signalling (Figure 1C; supplementary material, Table S6A).

Next, we interrogated global expression profiling data from BMP‐treated cells by using GSEA signatures specifically associated with colonic epithelial homeostasis and tumourigenesis (supplementary material, Table S6B1). Consistent with the putative homeostatic role of BMP signalling in the differentiating cells of the intestinal epithelium, we saw marked downregulation of genes associated with proliferation [BMP2 at 4 h, normalized enrichment score (NES) = –7.92, *p* < 0.001; BMP4 at 4 h, NES = –8.82, *p* < 0.001; BMP2 at 24 h, NES = –3.69, *p* = 0.002; BMP4 at 24 h, NES = –2.39, *p* = 0.028; supplementary material, Table S6B2]. We also noted statistically significant positive enrichment of gene signatures characterizing active Notch signalling (BMP2 at 4 h, NES = 4.37, *p* < 0.001; BMP4 at 4 h, NES = 3.94, *p* < 0.001; BMP2 at 24 h, NES = 2.46, *p* = 0.027; BMP4 at 24 h, NES = 2.74, *p* = 0.011; Figure 1D; supplementary material, Table S6B3) and EMT (BMP2 at 4 h, NES = 3.17, *p* = 0.003; BMP4 at 4 h, NES = 3.04, *p* = 0.002; BMP2 at 24 h, NES = 2.62, *p* = 0.011; BMP4 at 24 h, NES = 3.06, *p* = 0.002; Figure 1D; supplementary material, Table S6B4). Apoptosis and senescence were not significantly affected by BMP stimulation of immortalized HCECs (supplementary material, Table S6B5).

### BMP–Notch crosstalk in HCECs occurs through a TGF‐β‐independent mechanism and is differentially regulated by SMAD1 and SMAD5

**Figure 2:**
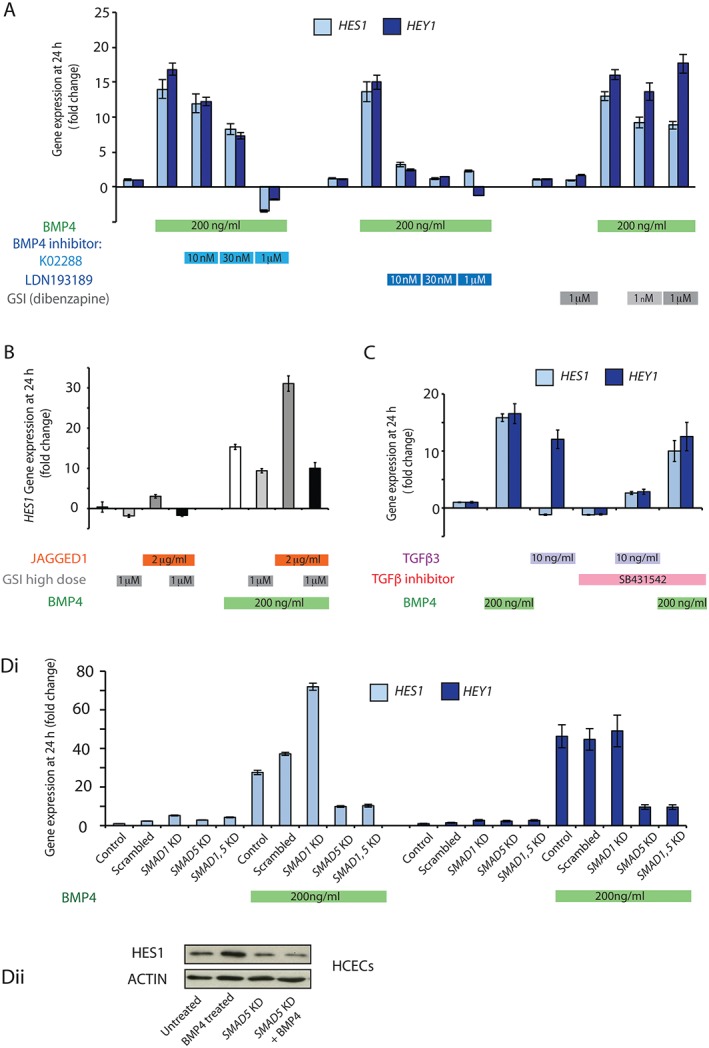
BMP4 induction of Notch target genes is independent of canonical Notch signalling and is regulated by SMAD proteins. (A) Relative HES1 and HEY1 mRNA levels in HCECs 24 h after control vehicle or BMP4 treatment with or without two different BMP inhibitors at three different concentrations (K02288 and LDN193189 at 10 nm, 30 nm and 1 µm) and two concentrations of the GSI dibenzazepine (1 nm and 1 µm). (B) Relative HES1 mRNA levels in HCECs 24 h after control vehicle, JAG1 and/or BMP4 stimulation, with or without γ‐secretase inhibition with 1 µm dibenzazepine. (C) Relative HES1 and HEY1 mRNA levels in HCECs 24 h after control vehicle, TGF‐β3 or BMP4 ligand stimulation, with or without the specific TGF‐β inhibitor SB431542. (Di) Relative HES1 and HEY1 mRNA levels in HCECs after SMAD1 or SMAD5 knockdown (KD) or simultaneous SMAD1 and SMAD5 KD) for 48 h followed by vehicle‐only control or BMP4 stimulation for 24 h. All values are mean ± standard error of the mean (n = 2). Differences in fold change in gene expression in KD cells are the consequence of increased cell density resulting from additional culture time in these experiments. (Dii) Western blot showing increased expression of HES1 in response to BMP4 (200 ng/ml) stimulation, and abrogation of this response following SMAD5 knockdown.

Given the enrichment of Notch signalling in response to BMP stimulation, we investigated the mechanism of BMP–Notch crosstalk in HCECs. At 1 µm, the selective BMP type 1 receptor inhibitor K02288 abolished recombinant BMP4 ligand induction of both BMP and Notch target genes, with dose‐dependent inhibition being seen at lower concentrations. In contrast, low concentrations (10–30 nm) of the specific ALK2/3 inhibitor LDN193189 effectively abrogated Notch target gene expression following BMP ligand stimulation. Given the selectivity of LDN193189 for BMP inhibition over TGF‐β or activin signalling, these results indicate that Notch signalling is downstream of receptor‐mediated activation of the BMP pathway rather than involving cross‐activation of TGF‐β or activin pathways 33 (Figure 2A; supplementary material, Figure S1). Similar results were obtained with BMP2 treatment (data not shown). Induction of *HES1* expression by BMP4 was partially abrogated by inhibition with the canonical Notch pathway γ‐secretase inhibitor (GSI), dibenzazepine (Figure 2A), whereas *HEY1* expression was unaffected. In contrast, γ‐secretase inhibition abolished *HES1* expression following Notch ligand (JAG1) stimulation (Figure 2B), indicating that BMP can activate Notch signalling through a γ‐secretase‐independent pathway. Interestingly, the combination of exogenous BMP4 and JAG1 stimulation had a synergistic effect on *HES1* upregulation, indicating that the biological effects of Notch activation could be amplified by the intracellular interaction between the BMP and Notch pathways (Figure 2B). To look for direct interactions between the BMP and Notch intracellular signalling pathways, we used ChIP with an anti‐SMAD1/5 antibody to pull down chromatin from BMP4‐treated HCECs. Enrichment for predicted regions of chromatin in the promoters of *HEY1* and *NOTCH1* indicated SMAD1/5 DNA binding. No strong evidence for SMAD binding was seen at our predicted binding sites in the *HES1* promoter; however, it is possible that binding occurs at upstream or downstream regions (supplementary material, Figure S1B). Together these data demonstrate an intracellular interaction between activated BMP signalling and the Notch pathway.

TGF‐β is known to activate Notch signalling 34, so, to exclude the effect of TGF‐β signalling as a potential intermediate pathway in this investigation, we assessed the effect of recombinant TGF‐β3 ligand stimulation on HCECs. TGF‐β activation had no effect on *HES1* expression but did upregulate *HEY1*, an effect that could be abrogated by co‐incubation with the TGF‐β receptor‐specific inhibitor SB431542. TGF‐β receptor inhibition had no significant effect on the activation of Notch target genes following BMP ligand stimulation, indicating that the crosstalk between the BMP and Notch pathways was not mediated through TGF‐β signalling (Figure 2C).

Next, we used siRNA to knock down BMP pathway constituents, to determine whether this affected downstream Notch activation. *SMAD5* knockdown (>50% knockdown; supplementary material, Figure S1E) suppressed BMP4 ligand activation of both *HES1* and *HEY1*, whereas *SMAD1* knockdown (>70% knockdown; supplementary material, Figure S1E) had no effect on *HEY1* expression and, surprisingly, markedly increased the *HES1* response to BMP4 ligand stimulation. The effect of combined simultaneous knockdown of *SMAD1* and *SMAD5* was similar to that seen with *SMAD5* knockdown alone. These results indicate that Notch target gene expression is variably regulated by SMAD proteins, with intact SMAD1 suppressing, and SMAD5 amplifying, *HES1* expression, following BMP4 stimulation; however, SMAD5 is the dominant mediator in determining the *HES1* response to BMP activation (Figure 2D).

### BMP and Notch signalling are partially segregated in normal colonic crypts but co‐localize in some human cancers

**Figure 3:**
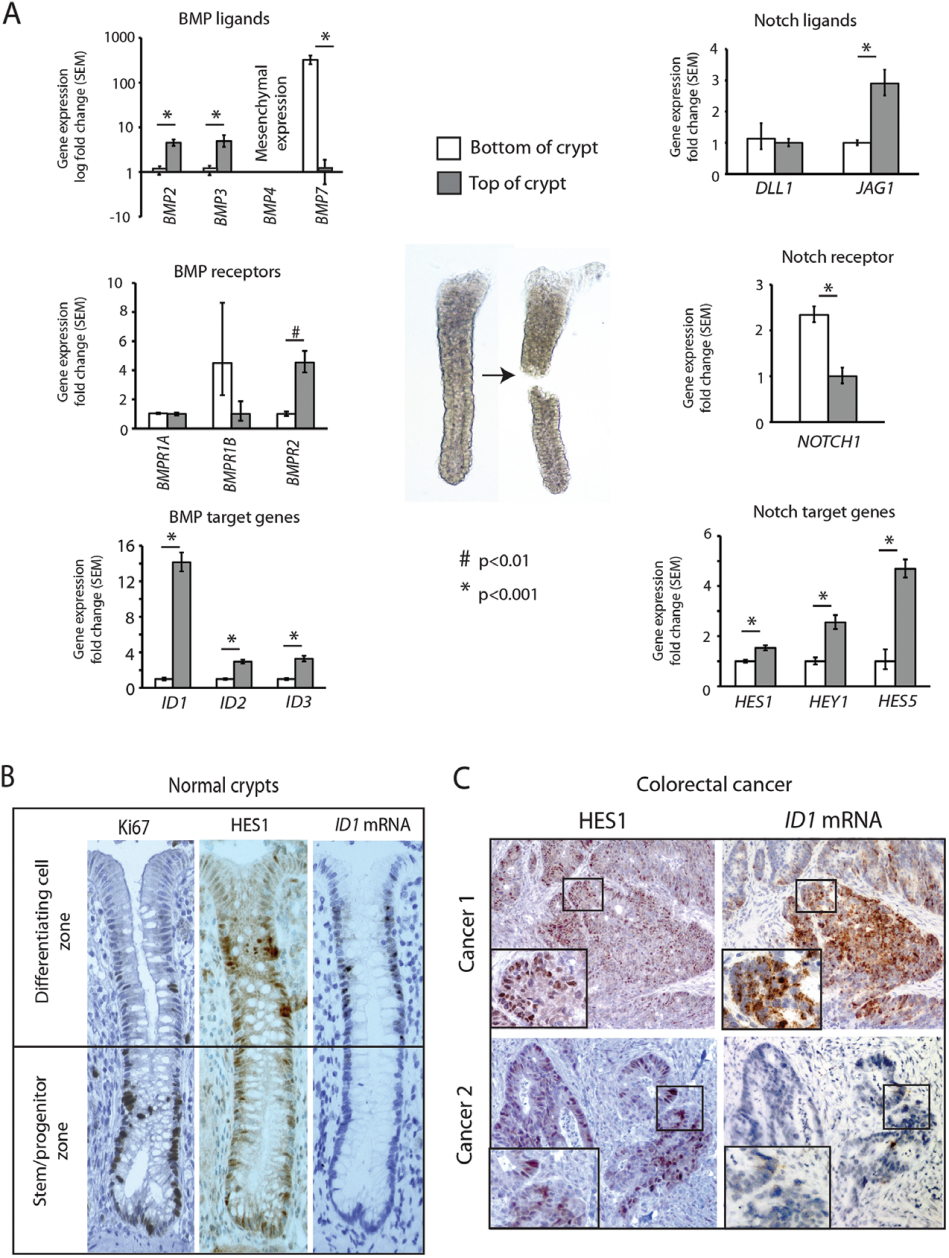
BMP and Notch signalling are partially segregated in normal colonic crypts but co‐localize in some human CRCs. (A) Relative mRNA expression levels of BMP and Notch pathway ligands, receptors and target genes in the bottom versus the top of hemisected individual normal human colonic crypts (n = 30, three patients). (B) Serial sections of normal human colonic tissue stained with Ki67‐specific and HES1‐specific antibodies and ID1 mRNA probe (magnification: ×200). (C) Serial sections of human colon tumours stained with HES1‐specific antibody and ID1 mRNA probe showing presence and co‐localization (Cancer 1) or differential expression (Cancer 2) of Notch and BMP pathway target genes, respectively, in CRCs. These are representative images from analysis of 105 paraffin‐embedded tumours. SEM, standard error of the mean.

Next, to ensure that BMP/Notch co‐expression and interaction was not a human cell line artefact, we extracted and hemisected individual normal human colonic crypts (30 from three patients) to assess the physiological expression of BMP and Notch pathway constituents and target genes. There was significantly higher expression of most BMP and Notch target genes in the top halves of colonic crypts, although *HES1* expression was more evenly spread across the crypt (Figure 3A). *In situ* hybridization for *ID1* and immunohistochemistry for HES1 and Ki67 on serial sections of human colonic crypts (54 well‐orientated crypts analysed from four paraffin blocks) showed co‐localization of pathway target genes in the cell population at the top of the crypt, with only *HES1* expression being seen in the dividing cells of the stem/progenitor zone (Figure 3B). Interestingly, the mRNA expression of the ligands and receptors of both pathways did not always mirror target gene activity, indicating the importance of mechanisms other than simple ligand–receptor interaction for regulating and segregating morphogen pathway activity (Figure 3A). These data indicate that, in the normal intestinal crypt, stringent control of morphogen signalling gradients partially segregates these pathways, with restricted co‐localization in the differentiating cell population and no evidence of shared expression in the stem/progenitor zone at the crypt base.

Next, we used *ID1* mRNA *in situ* hybridization and HES1 immunohistochemistry on serial sections from colorectal tumour tissue to assess whether loss of stringency in CRC resulted in more widespread co‐localization of these pathways. We saw considerable variability in *ID1* expression in human colorectal lesions, with positive staining in the malignant epithelium of 24% (25/105) of tumours, consistent with the known frequency of mesenchymal tumours (Figure 3C, cancer 1). Negligible *ID1* signals were seen in other samples (Figure 3C, cancer 2). Co‐localization of *ID1* and HES1 staining was seen in 76% (19/25) of the *ID1*‐positive samples (Figure 3C). BMP signalling principally acts upon the intestinal epithelial cell population 2. Consistent with this, we saw little or no *ID1*, p‐SMAD5 or HES1 expression in the stroma surrounding normal colonic crypts or CRCs (supplementary material, Figure S2A).

### The BMP signalling pathway activity is variable across different tumour subtypes

**Figure 4:**
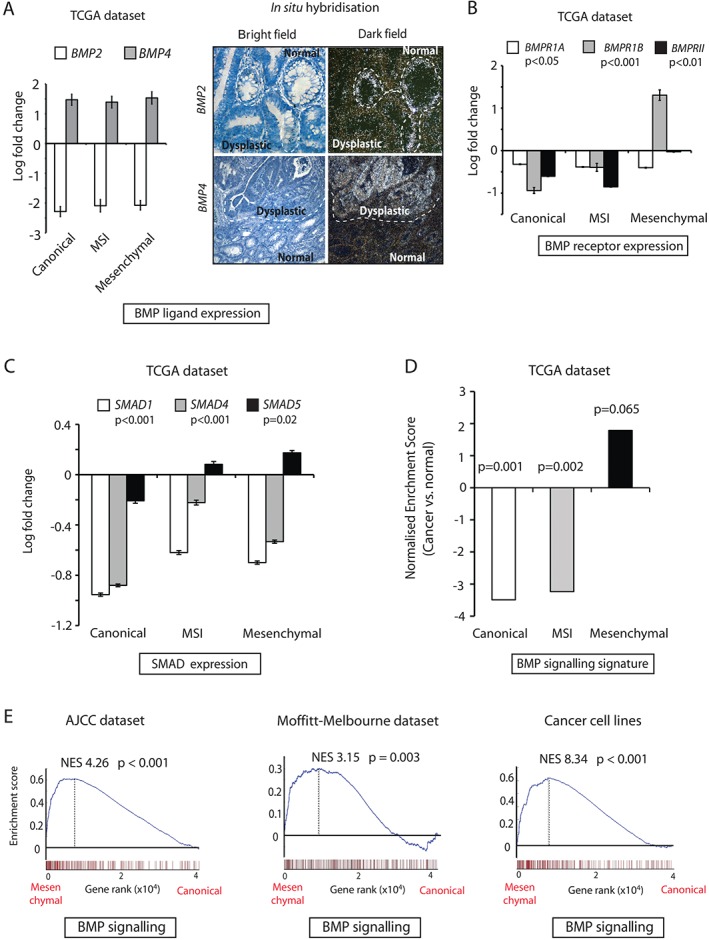
Variable BMP signalling in different CRC molecular subtypes. (A) Left: relative BMP2 and BMP4 ligand expression in TCGA CRC samples as compared with normal colonic tissues. CRC patients were divided into those having canonical (n = 149), MSI (n = 110) and mesenchymal (n = 78) tumours. Values are represented as log_2_ fold change ± standard error of the mean (SEM). P‐values were calculated with anova. Right: representative images of BMP2 and BMP4 mRNA in situ hybridization showing downregulation or upregulation of ligands, respectively, in dysplastic versus normal human colonic tissue. (B) Relative BMPR1A, BMPR1B and BMPRII mRNA expression in CRC samples as compared with normal colonic tissues. Values represent log_2_ fold change ± SEM. P‐values were calculated with anova. (C) Relative SMAD1, SMAD4 and SMAD5 mRNA expression in CRC samples as compared with normal colonic tissues. Values represent log_2_ fold change ± SEM. P‐values were calculated with anova. (D) Normalized enrichment scores on TCGA CRC subtypes obtained by use of an HCEC‐generated BMP signature (281 genes) as an a priori gene set. P‐values were generated with Kolmogorov–Smirnov statistics. (E) Gene set enrichment plots created by the use of a BMP signalling signature (281 genes) on two independent CRC datasets and a panel of CRC cell lines, comparing mesenchymal with canonical subtypes. P‐values were generated with Kolmogorov–Smirnov statistics.

Given the variable expression of mRNA of the BMP target gene *ID1* in the epithelium of different human CRCs, we assessed the mRNA expression levels of BMP signalling components in CRC subtypes by using TCGA RNA‐sequencing data 35. We saw differential expression of BMP2 and BMP4 ligands in all tumour subtypes, with downregulation of *BMP2* and upregulation of *BMP4* expression as compared with normal colon (Figure 4A). In intestinal homeostasis, BMP2 is expressed by both the mesenchyme and epithelium, whereas BMP4 is predominantly stromal in origin. We used isotopic *in situ* hybridization to show that the differential *BMP2*/*4* expression change identified in the TCGA dataset is predominantly the consequence of loss of normal *BMP2* expression and gain of aberrant *BMP4* expression in dysplastic intestinal epithelium (Figure 4A). *BMPR1A* expression was globally reduced across all CRC subtypes, but *BMPRII* expression was retained and *BMPR1B* expression was upregulated specifically in mesenchymal tumours (Figure 4B). Similarly, there was considerable variability in the expression of the *SMAD* genes. Reduced *SMAD1* and *SMAD4* expression was seen across all cancer subtypes, but *SMAD5* was differentially expressed: it was downregulated in canonical tumours (*n* = 149), but was retained and modestly upregulated in poor‐prognosis, mesenchymal tumours (*n* = 78) (Figure 4C). Immunohistochemistry confirmed variable epithelial expression of p‐SMAD5 in the malignant epithelium of different CRCs (supplementary material, Figure S2B). Moreover, we found that SMAD5 expression levels had independent prognostic value in stratifying patients with Stage 3 and 4 CRC into high‐risk and low‐risk groups (*p* = 0.046; supplementary material, Figure 2C).

As BMP4 appears to be the active ligand in CRC, we used our BMP4 transcriptional profiling data to generate a ‘BMP signature’ consisting of 281 genes with fold changes of >2 and FDRs of <0.05 in HCECs (supplementary material, Table S5B). We used this signature to investigate active BMP signalling activity across the different molecular subtypes of CRC in multiple publically available datasets. Using GSEA on TCGA RNA‐sequencing data, we found profound downregulation of BMP signalling in canonical tumours (*n* = 149, NES = –3.49, *p* = 0.001) as compared with normal colonic tissue (*n* = 41). Similarly, MSI tumours showed negative enrichment (*n* = 110, NES = –3.23, *p* = 0.002), but this trend was shifted towards upregulation of the BMP signature in mesenchymal tumours (*n* = 78, NES = 1.79, *p* = 0.065) (Figure 4D). Two independent datasets confirmed the BMP signature to be upregulated in the mesenchymal subtype as compared with the canonical subtype (Academic Medical Centre AJCC, NES = 4.26, *p* < 0.001; Melbourne–Moffitt, NES = 3.15, *p* = 0.003; Figure 4E) and MSI subtype (AJCC, NES = 4.10, *p* < 0.001; Melbourne–Moffitt, NES = 8.033, *p* < 0.001; supplementary material, Figure S2D). A panel of CRC cell lines classified into the CMS categories (canonical, *n* = 38; MSI, *n* = 14; and mesenchymal, *n* = 26) showed similar differential BMP signalling between mesenchymal tumours and other subtypes (mesenchymal versus canonical, NES = 8.34, *p* < 0.001; mesenchymal versus MSI, NES = 12.54, *p* < 0.001) (Figure 4E; supplementary material, Figure S2D).

### BMP–Notch crosstalk acts through SNAI1 to promote an EMT phenotype

**Figure 5:**
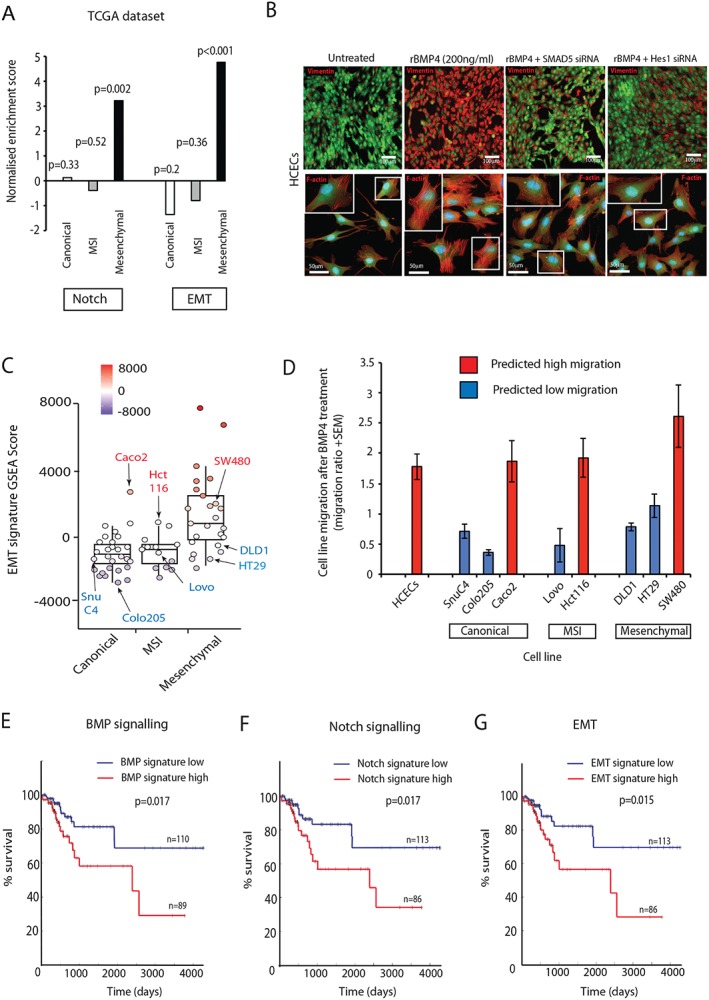
BMP–Notch interaction induces an EMT phenotype. (A) Normalized enrichment scores on TCGA CRC subtypes according to Notch and EMT signatures. P‐values were generated with Kolmogorov–Smirnov statistics. (B) Representative images of HCECs after 48 h of SMAD5 or HES1 knockdown and after 24 h of BMP4 treatment, stained for vimentin or F‐actin respectively. (C) Heterogeneity of EMT gene set enrichment analysis score in CRC lines, both within and between different tumour subtypes. (D) Cell migration ratio (cell migration after/before BMP4 stimulation) in a panel of predicted migratory and non‐migratory cancer cell lines. (E–G) Kaplan–Meier plots displaying recurrence‐free survival (RFS) over time in all AJCC stages in the TCGA dataset. Log‐rank test p‐value compares RFS over time for patients grouped by KNN clustering according to BMP signature (E), Notch signature (F), and EMT signature (G) (blue is below median expression levels of genes; red is above median expression levels).

BMP ligand stimulation of HCECs caused enrichment of genes associated with Notch signalling and EMT (Figure 1D). Notch signalling induces EMT 36, and together these pathways are associated with poor prognosis in patients with CRCs 37. We used GSEA analysis on the TCGA dataset to confirm that Notch and EMT gene signature enrichment mirrored the variable activation of our BMP signalling signature in CRC subtypes, with statistically significant upregulation in mesenchymal tumours only (Figure 5A). We postulated that BMP–Notch interaction can induce EMT in intestinal epithelial cells, and that this phenotype is promoted by the mesenchymal tumour microenvironment. To test this, we used reverse transcription quantitative PCR, and found a significant increase in expression of the EMT transcription factor genes *SNAI1* and *SLUG* in response to BMP4 stimulation of HCECs (supplementary material, Figure S3A). No effect was seen on *ZEB1* expression (data not shown). The effect on *SNAI1* induction was abrogated by disrupting the intracellular BMP and Notch pathways independently, by siRNA knockdown of *SMAD5* and *HES1*, respectively, but was unaffected by *HEY1* knockdown, suggesting that *SNAI1* is downstream of *HES1* alone in BMP–Notch crosstalk (supplementary material, Figure S3B). Consistent with our previous findings, *SMAD1* knockdown resulted in increased *SNAI1* expression, indicating differential regulation of the BMP–Notch pathway interaction by the different intracellular SMAD proteins (supplementary material, Figure S3B). BMP4 stimulation induced an EMT phenotype in HCECs, with increased expression of vimentin protein and dynamic remodelling of the actin cytoskeleton, as seen by assembly of F‐actin filaments into stress fibres, a phenomenon associated with migratory cells 38. Induction of this cellular phenotype was independently abrogated by siRNA disruption of mediating BMP and Notch signalling components (Figure 5B).

To functionally assess the EMT phenotype, we used cell migration assays to demonstrate a significant increase in HCEC motility following BMP4 stimulation (Figure 5D). Given the reported variable response of individual cancer cell lines to BMP stimulation 8, 14, 15, 16, we attempted to predict the migratory capacity of individual cancer cell lines before testing the effect of BMP4. By generating individual cell line EMT molecular signature scores, we could demonstrate considerable heterogeneity of cell line EMT gene signature expression, both within and between groups from different tumour subtypes (Figure 5C). We then assessed the effect of BMP4 stimulation on cell migration in a panel of cancer cell lines with a predicted migratory and non‐migratory line from each tumour subtype. This demonstrated that cancer cell lines with a molecularly defined EMT phenotype are responsive to BMP4‐induced cell migration, irrespective of tumour subtype gene signature (Figure 5D).

### BMP, Notch and EMT gene signatures independently identify patients with high‐risk or low‐risk CRC

To investigate the prognostic implications of a mechanistic link between BMP–Notch interaction and induction of an EMT phenotype, we analysed associations between our BMP signalling signature (281 genes), Notch target genes (83 genes) 28 and curated EMT gene signature (84 genes) 27 with recurrence‐free survival in the TCGA dataset. The three gene signatures are distinct, with <10% overlap between them (supplementary material, Figure S3C), but, within individual tumours in the TCGA cohort, there was a significant correlation between expression levels of BMP, Notch and EMT signatures (Fisher exact test, *p* < 0.01; supplementary material, Figure S3D). Importantly, patients with above‐median expression of each individual gene signature had significantly shorter disease‐free survival, with striking similarities being seen between the Kaplan–Meier curves for the different gene sets (BMP, *p* = 0.017; Notch, *p* = 0.017; EMT, *p* = 0.015; Figure 5E–G). This demonstrates that BMP, Notch and EMT gene signatures have similar prognostic capabilities, and supports the involvement of a BMP–Notch–EMT axis in the biological mechanisms that contribute to poor prognosis in CRC.

## Discussion

**Figure 6:**
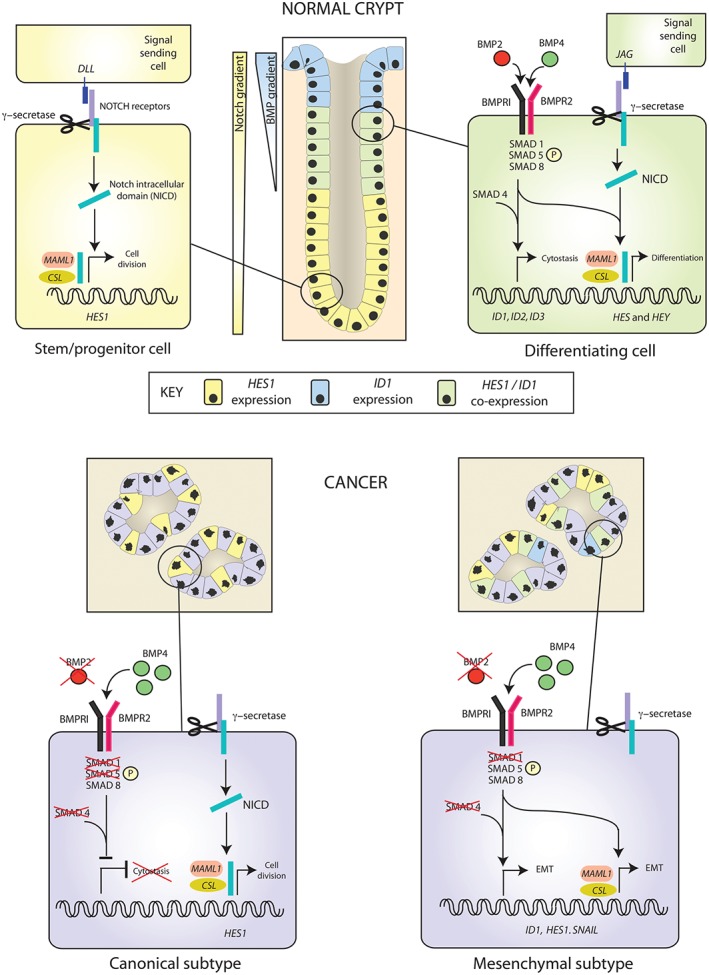
Model of BMP–Notch interaction in intestinal homeostasis and CRC disease subtypes. In intestinal homeostasis, the BMP and Notch pathways are partially segregated. In the stem/progenitor zone at the base of the crypt, Notch signalling regulates intestinal stem cells and promotes progenitor cell division. Specific co‐localization of BMP and Notch signalling in cells emerging from the transit‐amplifying zone inhibits cell proliferation and promotes differentiation. In canonical tumours, loss of canonical BMP pathway constituents downregulates BMP signalling, promoting cell proliferation. In mesenchymal tumours, impaired pathway segregation, loss of SMAD1 and retention of SMAD5 enhance BMP–Notch interaction. In this model, BMP and Notch pathways act synergistically via a γ‐secretase‐independent mechanism to promote an EMT phenotype, which we postulate is promoted by the mesenchymal tumour microenvironment.

The BMP and Notch pathways are important, highly conserved morphogenetic signalling pathways with many convergent physiological functions in different tissue systems. It was previously assumed that these morphogens acted in parallel, independently regulating transcription of their own target genes 39; however, more recently, synergistic or antagonist pathway interaction has been described in diverse tissue contexts, including the mouse eye ciliary bodies 40, skeletal muscle stem cells 41, and vascular endothelium 42. In this study, we demonstrate, for the first time, that synergistic BMP and Notch signalling crosstalk also occurs in human intestinal epithelial cells, and show that this interaction can be regulated at an intracellular level, as differential *SMAD* knockdown amplified or attenuated the Notch pathway response to BMP stimulation. In the homeostatic intestinal epithelial microenvironment, morphogen signalling activity is necessarily strictly regulated, with target gene activity being segregated to different functional compartments of the crypt. BMP and Notch co‐activation is restricted to the upper part of the human intestinal colonic crypt, and we hypothesize that the physiological role of pathway crosstalk is to translate the paracrine effect of secreted BMP ligands into cell‐to‐cell, binary cell fate decisions through Notch signalling, inducing terminal differentiation of cells as they exit the transit‐amplifying zone. Further *in vivo* experiments will be required to confirm this (Figure 6).

In cancer, signalling dysregulation disrupts the finely controlled morphogen balance and pathway segregation. Genetic context and differential expression of BMP ligands, receptors, intracellular signalling proteins and antagonists in human cancers and CRC cell lines has led to confusion over the functional role and therapeutic potential of BMP signalling. In this study, we used normal explant‐derived intestinal epithelial cells to generate a functional signature that characterizes BMP signalling. We used this to demonstrate variably active BMP signalling across different CRC subtypes and cell lines. Consistent with an anti‐proliferative homeostatic role, BMP signalling is actively downregulated in the majority of CRCs, but is paradoxically conserved in poor‐prognosis, mesenchymal tumours, where it acts through Notch signalling to promote an EMT phenotype (Figure 6). This demonstration of BMP signalling heterogeneity across different cancer subtypes and cell lines may help to account for previous *in vitro* experimental inconsistencies 12, 13, 15, 16, 17. Part of this variability may be explained by retained expression of SMAD5, which is the key intracellular mediator between the BMP and Notch pathways, in the intestinal epithelium in mesenchymal tumours.

Like any investigation of human cell biology, this study has its limitations. Our experimental model required the use of a human cell line derived from a normal adult intestinal explant. These cells are diploid and have intact BMP and Notch signalling components, but have been immortalized by expression of *cyclin dependent kinase 4* (*CDK4*) and *catalytic component of human telomerase* (*hTERT*) 21. Primary human intestinal organoids were considered, but are dependent on Noggin supplementation and are unable to tolerate BMP ligand stimulation, which is cytostatic and induces organoid collapse. There is a risk of generating artefactual results from any *in vitro* assessment of signalling pathways that are normally dependent on intercompartmental cellular interaction; however, the use of a normal explant‐derived line over CRC cell lines to assess BMP signalling is justified by the recently demonstrated influence of genetic mutation on BMP pathway activity 18, 19. Furthermore, we have generated a clinically relevant BMP signalling signature that has prognostic implications in human CRC.

Our increasing understanding of the dysregulated molecular signalling pathways in cancer has provided a platform for the development of targeted therapies. The development of BMP‐targeted agents has been hindered by confusion over the biological role of the pathway in cancer 43. Dysregulated Notch signalling has been implicated in many cancers, where it has a tumour‐initiating 44, tumour‐promoting or tumour suppressor 45 function, depending on cellular context and interactions with other pathways. In CRC, increased expression of Notch pathway constituents has been demonstrated in mouse intestinal tumour models 46, human cell lines 47, and some intestinal tumours 48. Furthermore, pharmacological inhibition of the canonical Notch signal with GSIs prompted secretory differentiation of proliferative adenoma cells and a 50% reduction in *Apc^Min^* mouse tumour burden 46. GSIs have been proposed as a potential therapy for colon cancer 49, and have undergone numerous phase 1 combination chemotherapy trials in advanced solid tumours 50, 51. Our work shows that the inhibition of canonical Notch signalling in mesenchymal CRCs is unlikely to be effective, as it will not prevent direct Notch activation by BMP signalling, which acts through a γ‐secretase‐independent mechanism. Differential therapeutic targeting of CRC based on molecular subtype is an exciting prospect; however, further research is required to understand the cellular mechanisms that underpin the variable biology of different CRC types. This study, identifying a synergistic interaction between two potentially targetable signalling pathways in aggressive CRCs, highlights the requirement for multiple therapeutic agents to address the complex signalling derangement in CRC.

## Author contributions statement

The authors contributed in the following way: SI, SJL: conceived and designed the project; SI, HD, AAZ, BB, CMAP, HR, CB, AL, SJL: conducted experiments; RJ: completed isotopic *in situ* hybridization; SB: collected human tissue; SI, MB, PG: carried out bioinformatic analysis; LMW, JEE: pathology support, tissue provision, and intellectual input; SI, SJL: wrote the manuscript; IT: critically analysed the manuscript.

**SUPPLEMENTARY MATERIAL ONLINE**

**Supplementary materials and methods**

**Supplementary figure legends**

**Figure S1.** Relative mRNA expression levels of BMP and Notch target genes, and protein levels of SMAD1 and SMAD5 in HCECs

**Figure S2.** Variable BMP and Notch signalling in different colorectal cancer molecular subtypes

**Figure S3.** Relative mRNA expression levels of *SNAIL1* and *SLUG* in HCECs and overlap of BMP, Notch and EMT signatures in primary human colorectal cancers

**Table S1.** Primers used in chromatin immunoprecipitation experiments

**Table S2A.** VSN normalised gene expression profiling data from HCECs either vehicle‐only for 4 h (*n* = 3), 200 ng/ml BMP2‐treated for 4 h (*n* = 3) or 200 ng/ml BMP4‐treated for 4 h (*n* = 3)

**Table S2B.** VSN normalised gene expression profiling data from HCECs either vehicle‐only for 24 h (*n* = 3), 200 ng/ml BMP2‐treated for 24 h (*n* = 3) or 200 ng/ml BMP4‐treated for 24 h (*n* = 3)

**Table S3A.** Differentially expressed genes between 200 ng/ml BMP2‐treated HCECs for 4 h (*n* = 3) and untreated cells (*n* = 3) compared to vehicle‐only HCECs (*n* = 3)

**Table S3B.** Differentially expressed genes between 200 ng/ml BMP2‐treated HCECs for 24 h (*n* = 3) and untreated cells (*n* = 3) compared to vehicle‐only HCECs (*n* = 3)

**Table S4A.** Differentially expressed genes between 200 ng/ml BMP4‐treated HCECs for 4 h (*n* = 3) and vehicle‐only HCECs (*n* = 3)

**Table S4B.** Differentially expressed genes between 200 ng/ml BMP4‐treated HCECs for 24 h (*n* = 3) and vehicle‐only HCECs (*n* = 3)

**Table S5A.** Overlapping differentially expressed genes induced by 200 ng/ml BMP2 or 200 ng/ml BMP4 after 4 h of treatment

**Table S5B.** Overlapping differentially expressed genes induced by 200 ng/ml BMP2 or 200 ng/ml BMP4 after 24 h of treatment

**Table S6A.** Overlapping upregulated pathway enrichment using C2 Broad Institute database 32 induced by 200 ng/ml BMP2 or 200 ng/ml BMP4 treatment in human colonic epithelial cells

**Table S6B1.** List of *a priori* gene signatures used in interrogating gene expression data from BMP2/4‐treated HCECs using Gene Set Enrichment Analysis

**Table S6B2.** GSEA proliferation results

**Table S6B3.** GSEA Notch results

**Table S6B4.** GSEA epithelial–mesenchymal transition results

**Table S6B5.** GSEA apoptosis results

## Supporting information


**Supplementary Materials and Methods**

**Supplementary figure legends**

**Figure S1.** Relative mRNA expression levels of BMP and Notch target genes, and protein levels of SMAD1 and SMAD5 in HCEC cells. **1A)** Relative mRNA expression of BMP and Notch target genes in HCEC cells 4 and 24 h after vehicle only (control) or BMP4 stimulation. **1B)** Relative enrichment as determined by the ΔΔCt method using sonicated input DNA to normalize the values from immunoprecipitated DNA and expressing this ratio relative to that seen at the GAPDH promoter as a negative control. **1C)** Relative ID1, ID2 and ID3 mRNA levels in HCEC cells 24 h after control vehicle only or BMP4 treatment, +/‐ two different BMP inhibitors (K02288 and LDN193189) **1D)** Relative ID1, ID2 and ID3 mRNA levels in HCEC 24 h after control vehicle or BMP4 treatment, +/‐ two concentrations of the γ‐secretase inhibitor (GSI), dibenzazepine. **1Ei)** Relative mRNA expression of SMAD1 and 5 genes in HCEC cells 72 h after SMAD 1 or 5 and simultaneous SMAD 1 and 5 knockdown with siRNA. **1Eii)** Western blot showing 80% knockdown SMAD1 and 50% knockdown of SMAD5 (quantification not shown) **1F)** Relative ID1 and ID3 mRNA levels in HCEC cells after SMAD1, 5 or simultaneous SMAD 1 and 5 knockdown (KD) for 48 h followed by vehicle control or BMP4 stimulation for 24 h. All values are mean ± SEM.

**Figure S2.** Variable BMP and Notch signalling in different colorectal cancer molecular subtypes. **2A)** Representative images of HES1, ID1 mRNA and p‐SMAD5 staining in human colorectal cancer samples showing staining predominantly restricted to the epithelial compartment with little or no stromal staining. **2B)** Representative images of p‐SMAD5 staining in human colon tumours from a tissue microarray (n = 105) **2C)** Kaplan‐Meier plot displaying recurrence‐free survival (RFS) over time in stage III and IV patients from the TCGA cohort. Log rank test p‐value compares RFS over time for patients grouped by KNN clustering according to SMAD5 expression levels. **2D)** Gene set enrichment plots using 281 BMP signalling signature on two independent CRC datasets and a panel of colorectal cancer cell lines comparing mesenchymal versus MSI subtypes. P‐values generated by Kolmogorov–Smirnov statistics.

**Figure S3.** Relative mRNA expression levels of SNAIL1 and SLUG in HCEC cells and overlap of BMP, Notch and EMT signatures in primary human colorectal cancers. **3A)** Relative SNAI1 and SLUG mRNA levels in HCEC cells, 24 h after BMP4 treatment compared to control cells. Values are mean ± SEM (n = 2). **3B)** Relative SNAI1 expression levels (fold change) in HCEC cells, 48 h after SMAD1, 5, HES1 or HEY1 knockdown (KD) and after 24 h of BMP4 treatment compared to control cells. Values are mean ± SEM (n = 2). **3C)** Venn diagrams showing overlap of genes in generated BMP signature and curated Notch and EMT signatures. WNT5A is the only gene common to all signatures. **3D)** Overlap of above and below median expression of BMP signalling, Notch and EMT gene signatures in numbers of different tumours in the TCGA dataset (Fishers exact test, p < 0.01).

**Table S1.** Primers used in chromatin immunoprecipitation experiments

**Supplementary Table 2A:** VSN normalised gene expression profiling data from HCECs either vehicle‐only for 4 h (n = 3), 200 ng/ml BMP2‐treated for 4 h (n = 3) or 200 ng/ml BMP4‐treated for 4 h (n = 3)

**Supplementary Table 2B:** VSN normalised gene expression profiling data from HCEC cells either untreated for 24h (n=3), 200ng/ml BMP2‐treated for 24h (n=3) or 200ng/ml BMP4‐treated for 24h (n=3)

**Supplementary Table 3A:** Differentially expressed genes between 200ng/ml BMP2 treated HCECs for 4h (n=3) and untreated cells (n=3)

**Supplementary Table 3B:** Differentially expressed genes between 200ng/ml BMP2 treated HCECs for 24h (n=3) and untreated cells (n=3)

**Supplementary Table 4A:** Differentially expressed genes between 200ng/ml BMP4 treated HCEC cells for 4h (n=3) and untreated cells (n=3)

**Supplementary Table 4B:** Differentially expressed genes between 200ng/ml BMP4 treated HCEC cells for 24h (n=3) and untreated cells (n=3)

**Supplementary Table 5A:** Overlapping differentially expressed genes induced by 200ng/ml BMP2 or 200ng/ml BMP4 after 4h of treatment

**Supplementary Table 5B:** Overlapping differentially expressed genes induced by 200ng/ml BMP2 or 200ng/ml BMP4 after 24h of treatment

**Supplementary Table 6A:** Overlapping, upregulated pathway enrichment using C2 Broad Institute database^30^ induced by 200ng/ml BMP2 or 200ng/ml BMP4 treatment in HCECs

**Supplementary Table 6B:** Detailed Gene Set Enrichment results using BMP2/4 induced gene expression data on various gene signatures

**Supplementary Table 6B1:** List of a priori gene signatures used in interrogating gene expression data from BMP2/4 treated HCEC cells using Gene Set Enrichment Analysis

**Supplementary Table 6B2:** Proliferation signature

**Supplementary Table 6B3:** Notch_Signalling_Target

**Supplementary Table 6B4:** EMT signature

**Supplementary Table 6B5:** Apoptotic signature
