# Supplementary material for: Bone morphogenetic protein and Notch signalling crosstalk in poor‐prognosis, mesenchymal‐subtype colorectal cancer
Source: J Pathol. 2017 May 3;242(2):178–92. doi: 10.1002/path.4891 (PMC5488238; doi:10.1002/path.4891)
Supplement: Supplementary file 1 — Supplementary Materials and Methods [file PATH-242-178-s001.docx]

**Supplementary Materials and Methods**

**Recombinant protein treatment of human colonic epithelial cells.** Cells were grown in serum free medium for 16 h, followed by treatment with 200 ng/ml recombinant human BMP2/BMP4, 10ng/ml human TGFB3 or 2 μg/ml rhJagged1 (R&D Systems) in 2% serum containing medium for 4 or 24 h. Cells treated with 4 mM HCl containing 0.1% bovine serum albumin or PBS were used as controls.

**Treatment of human colonic epithelial cells with BMP/γ-secretase/TGFβ inhibitors.** HCEC cells were plated at 70 % confluency, grown in serum free medium for 16 h and treated for 24 h in 2% serum containing medium supplemented with variable doses of BMP type 1 receptor inhibitors, K02288 or LDN-193189 (kind gift from Dr Alex Bullock, Oxford University), γ-secretase inhibitor, dibenzazepine (Merck Millipore) or 10 μM TGF-β Type 1 receptor kinase inhibitor, SB -431542 (Sigma-Aldrich). DMSO-treated cells were used as controls.

**Knockdown of SMAD1, 5, HES1, HEY1 in human colonic epithelial cells.** We used reverse transfection method using HiPerfect transfection reagent (Qiagen) to knockdown SMAD1 and SMAD5 (as these are the SMAD proteins mediating BMP/Notch interaction in muscle cells [41] and vascular endothelium [42], HES1, and HEY1. We did not assess the effect of SMAD4 knockdown as this protein is common to both TGF and BMP pathways and the effect of SMAD4 loss has been previously explored [19]. We seeded 6 X 10^4^ cells per well of a 24-well plate in 0.1 ml of growth medium containing serum and antibiotics. We diluted siRNA (FlexiTube siRNA, Qiagen), in culture medium without serum to a final concentration of 10 nM. Cells were incubated with transfection complexes for 48-72h. All Star Negative Control siRNA (SI03650318, Qiagen) was used as controls. Knockdown was confirmed with RT-qPCR and Western Blot (Supplementary Figure S1D).

**Immunohistochemistry.** Formalin-fixed, paraffin-embedded sections (4 µm) were de-waxed in xylene and rehydrated through graded alcohols to water. Endogenous peroxidase was blocked using 0.3% hydrogen peroxide for 20 min. For antigen retrieval, sections were pressure cooked in 10 mmol/L citrate buffer (pH 6.0) for 5 min at 95- 100 °C. Sections were blocked with 5% serum for 1 h. Slides were incubated with primary antibody overnight at 4 °C. The following antibodies have been used in this study: Ki67 (human) (DAKO, MIB-1), Anti-SMAD5 phospho S463 + S465 (Abcam, ab92698), HES1 (Cell Signaling Technology, D6P2U, #11988). Appropriate secondary antibodies were applied for 1 h at room temperature. Sections were then incubated in ABC (Vector labs) for 30 min. DAB solution (Sigma) was applied for 2–15 min.

***In situ* hybridisation (ISH).** 4 µm sections were prepared using DEPC (Sigma)-treated H_2_0.  *In situ* hybridisation was carried out using the ID1 (414351), PPIB (313901), and DapB (310043)(Advanced Cell Diagnostics) probe sets and the RNAscope 2.5 HD Detection Kit (Advanced Cell Diagnostics) following the manufacturer’s instructions. For isotopic *in situ* hybridisation exon-spanning riboprobes were designed for human *BMP2* and *BMP4* and a *β-actin* probe was used for hybridization control. Riboprobes were generated by *in vitro* transcription using SP6 polymerase and labelled with S^35^-UTP (GE Healthcare, Chalfont St Giles, UK). Other methods, as described by Poulsom *et al*. [23]

**Immunofluorescence.** Cells were incubated in serum free medium supplemented with 0.1 μM BD Pharmingen^TM^ Calcein AM dye for 30 mins at 37 °C protected from light. Cultures were washed, followed by cell fixation with 4% paraformaldehyde in PBS for 15 min at room temperature. This was followed by washing and blocking in 1X PBS /5% normal serum /0.3% Triton™ X-100 for 1 h at room temperature. Blocking buffer was aspirated and cells were incubated with primary antibody overnight at 4 °C. Primary antibodies used in this study were Vimentin (Cell Signaling Technology, D21H3 #5741) and Alexa Fluor**®** 647 Phalloidin (Cell Signaling Technology, #8940). Cells were then rinsed and incubated in fluorochrome-conjugated secondary antibody (Alexa Fluor**®** 488, ThermoFisher Scientific) for 1 h at room temperature in the dark, followed by counterstaining with DAPI (ThermoFisher Scientific). Cells were visualized using a Operetta High-Content fluorescence microscope (Perkin Elmer).

**Gene expression arrays.** Raw gene expression values were normalized using the VSN (variance-stabilisation and normalisation) function from lumi package in bioconductor [5] (Supplementary Table S1). We applied a filter by taking a detection score of > 0.95 of the background intensity distribution for all samples to consider a probe detectable. Differentially expressed genes between experimental groups (rhBMP2 or rhBMP4 treated for 4 h (n=3) and 24 h (n=3) and control (vehicle treated, 4 mM HCl containing 0.1% bovine serum albumin, for 4 h (n=3) and 24 h (n=3)) were identified using Student’s *t*-test by running “ttest2” command in MATLAB® (Supplementary Table S2).

**Gene Set Enrichment Analysis (GSEA)**. GSEA was performed as described [24]. We computed the differential expression of genes in the experimental group versus control samples, and then used the t-statistics as a ranking metric. If multiple probes were present for a gene, probe with the highest absolute differential expression between experimental and normal was selected. We set gene shuffling at 1,000 permutations to compute the *P-*value for the enrichment score. A list of gene signatures [25-28] used in the enrichment analysis is given in Supplementary Table S5B1. If the signature was from a mouse dataset, we mapped these mouse genes to their human orthologs using the sequence-based method available from MGI (http://www.informatics.jax.org/). For EMT signature analysis in individual colorectal cancer cell lines, we used single sample GSEA.

**Chromatin immunoprecipitation**

ChIP assays were carried out as previously described [22]. In brief, approx.. 10^7^ cells were crosslinked for 10 min with 1% formaldehyde, neutralized with 125 mM glycine, scraped and washed with PBS. Lysis was carried out in 1% SDS, 10 mM EDTA, 50 mM Tris-HCl, and the chromatin sheared by sonication. (Bioruptor for 7-15 x 15 s cycles). Immunoprecipitations were carried out in 1% Triton-100, 2 mM EDTA, 150 mM NaCl, 20 mM Tris using 10 µg of anti-SMAD1/5 antibody (BMR00479 Bio Matrix Research) overnight at 4 °C. Protein A Dynabeads (Invitrogen) were used to capture the antibody/chromatin complexes which were washed with TSEI (0.1% SDS, 1% TritonX-100, 2 mM EDTA, 20 mM Tris, 150 mM NaCl), TSEII (0.1% SDS, 1% TritonX-100, 2 mM EDTA, 20 mM Tris, 500 mM NaCl) , LiCl buffer (0.25 M LiCl, 1% NP-40, 1% deoxycholate, 1 mM EDTA, 10 mM Tris-HCl) and TE according to standard protocols, and eluted with 1% SDS, 0.1 M NaHCO_3_. 1 µl of DNA was analysed in duplicate by SYBR green qPCR using the described primers (Supplementary Table S1).

**Cell migration analysis**

Colorectal cancer cell lines were grown in serum deprived medium overnight. 100 μl of full growth medium was added to 0.8 μm Transwell inserts and 600 µl in receiver wells of Transwell plates (Corning). The plates were then incubated for at least one hour in cell culture incubator. Cells were trypsinised, counted and plated at 3x10^5^ cells per well with either 200 ng/ml of recombinant human BMP4 protein (R and D Systems) or vehicle only BSA-HCl control. After 48 h, cells that had not migrated through the Transwell were removed while migrated cells were fixed in 4% paraformaldehyde and stained with DAPI. The 0.8 μm membrane was cut, mounted onto a microscope slide and coverslipped. DAPI-positive cells, representing migrated cells, were counted under a confocal microscope. Each condition was repeated in triplicates for each cell line in two independent experiments.
