# Supplementary material for: Bone morphogenetic protein and Notch signalling crosstalk in poor‐prognosis, mesenchymal‐subtype colorectal cancer
Source: J Pathol. 2017 May 3;242(2):178–92. doi: 10.1002/path.4891 (PMC5488238; doi:10.1002/path.4891)
Supplement: Supplementary file 2 — Supplementary figure legends [file PATH-242-178-s021.docx]

**Supplementary Figure Legends**

**Figure S1** Relative mRNA expression levels of BMP and Notch target genes, and protein levels of SMAD1 and SMAD5 in HCEC cells

**1A)** Relative mRNA expression of BMP and Notch target genes in HCEC cells 4 and 24 h after vehicle only (control) or BMP4 stimulation. **1B)** Relative enrichment as determined by the ΔΔCt method using sonicated input DNA to normalize the values from immunoprecipitated DNA and expressing this ratio relative to that seen at the *GAPDH* promoter as a negative control. **1C)** Relative *ID1, ID2* and *ID3* mRNA levels in HCEC cells 24 h after control vehicle only or BMP4 treatment, +/- two different BMP inhibitors (K02288 and LDN193189) **1D)** Relative *ID1*, ID2 and *ID3* mRNA levels in HCEC 24 h after control vehicle or BMP4 treatment, +/- two concentrations of the γ-secretase inhibitor (GSI), dibenzazepine. **1Ei)** Relative mRNA expression of *SMAD1* and *5* genes in HCEC cells 72 h after *SMAD 1* or *5* and simultaneous *SMAD 1* and *5* knockdown with siRNA. **1Eii)** Western blot showing 80% knockdown SMAD1 and 50% knockdown of SMAD5 (quantification not shown) **1F)** Relative *ID1* and *ID3* mRNA levels in HCEC cells after *SMAD1, 5* or simultaneous *SMAD 1* and *5* knockdown (KD) for 48 h followed by vehicle control or BMP4 stimulation for 24 h. All values are mean ± SEM.

**Figure S2.** Variable BMP and Notch signalling in different colorectal cancer molecular subtypes

**2A)** Representative images of HES1, *ID1* mRNA and p-SMAD5 staining in human colorectal cancer samples showing staining predominantly restricted to the epithelial compartment with little or no stromal staining. **2B)** Representative images of p-SMAD5 staining in human colon tumours from a tissue microarray (n=105) **2C)** Kaplan-Meier plot displaying recurrence-free survival (RFS) over time in stage III and IV patients from the TCGA cohort. Log rank test p-value compares RFS over time for patients grouped by KNN clustering according to *SMAD5* expression levels. **2D)** Gene set enrichment plots using 281 BMP signalling signature on two independent CRC datasets and a panel of colorectal cancer cell lines comparing mesenchymal versus MSI subtypes. P-values generated by Kolmogorov–Smirnov statistics.

**Figure S3** Relative mRNA expression levels of *SNAIL1* and *SLUG* in HCEC cells and overlap of BMP, Notch and EMT signatures in primary human colorectal cancers.

**3A)** Relative *SNAI1* and *SLUG* mRNA levels in HCEC cells, 24 h after BMP4 treatment compared to control cells. Values are mean ± SEM (n = 2). **3B)** Relative *SNAI1* expression levels (fold change) in HCEC cells, 48 h after *SMAD1*, *5*, *HES1* or *HEY1* knockdown (KD) and after 24 h of BMP4 treatment compared to control cells. Values are mean ± SEM (n = 2). **3C)** Venn diagrams showing overlap of genes in generated BMP signature and curated Notch and EMT signatures. *WNT5A* is the only gene common to all signatures. **3D)** Overlap of above and below median expression of BMP signalling, Notch and EMT gene signatures in numbers of different tumours in the TCGA dataset (Fishers exact test, p<0.01).
