# Supplementary figures and images for: Bone morphogenetic protein and Notch signalling crosstalk in poor‐prognosis, mesenchymal‐subtype colorectal cancer
Source: J Pathol. 2017 May 3;242(2):178–92. doi: 10.1002/path.4891 (PMC5488238; doi:10.1002/path.4891)

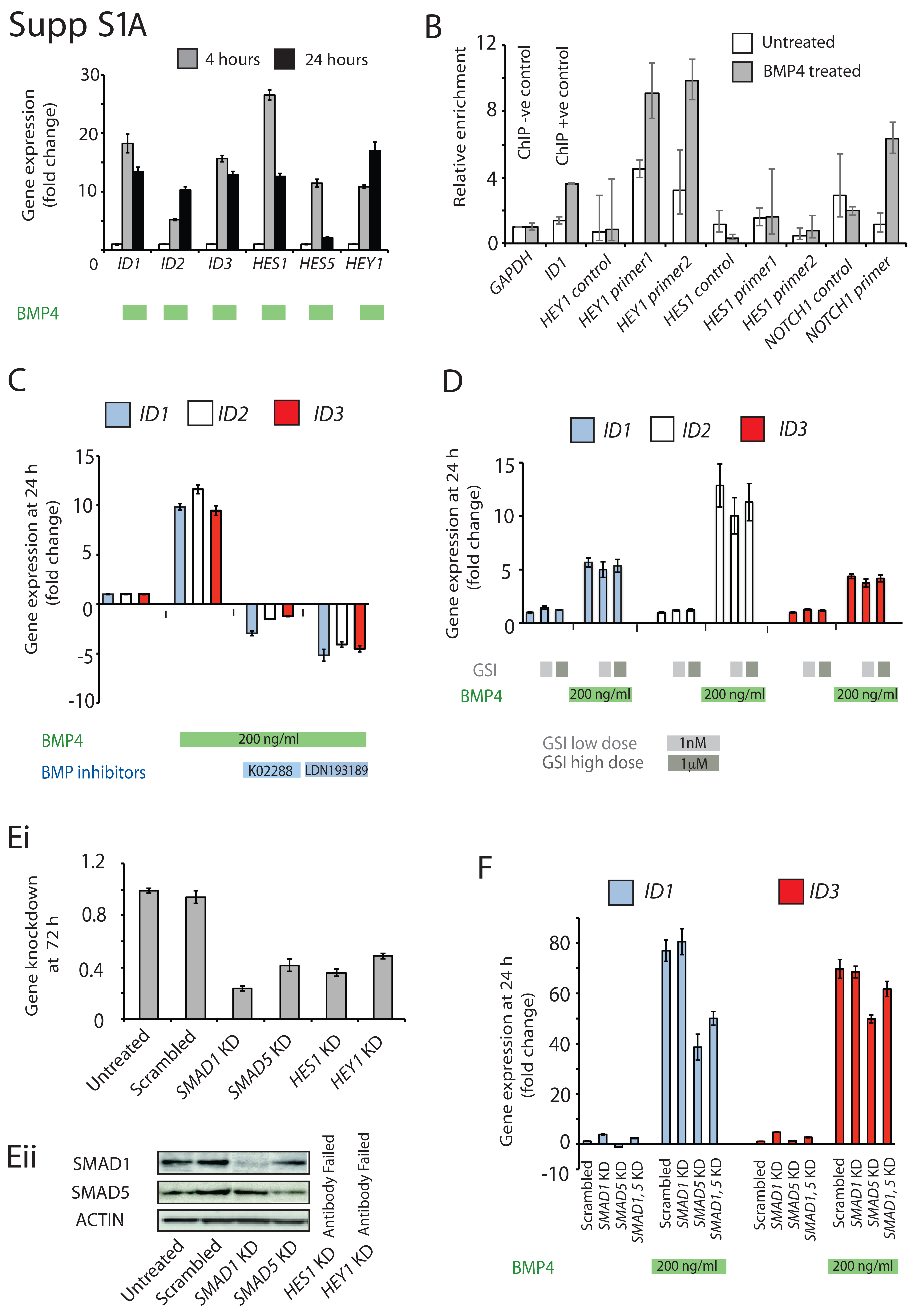

Supplement: Supplementary file 3 — Figure S1. Relative mRNA expression levels of BMP and Notch target genes, and protein levels of SMAD1 and SMAD5 in HCEC cells. 1A) Relative mRNA expression of BMP and Notch target genes in HCEC cells 4 and 24 h after vehicle only (control) or BMP4 stimulation. 1B) Relative enrichment as determined by the ΔΔCt method using sonicated input DNA to normalize the values from immunoprecipitated DNA and expressing this ratio relative to that seen at the GAPDH promoter as a negative control. 1C) Relative ID1, ID2 and ID3 mRNA levels in HCEC cells 24 h after control vehicle only or BMP4 treatment, +/‐ two different BMP inhibitors (K02288 and LDN193189) 1D) Relative ID1, ID2 and ID3 mRNA levels in HCEC 24 h after control vehicle or BMP4 treatment, +/‐ two concentrations of the γ‐secretase inhibitor (GSI), dibenzazepine. 1Ei) Relative mRNA expression of SMAD1 and 5 genes in HCEC cells 72 h after SMAD 1 or 5 and simultaneous SMAD 1 and 5 knockdown with siRNA. 1Eii) Western blot showing 80% knockdown SMAD1 and 50% knockdown of SMAD5 (quantification not shown) 1F) Relative ID1 and ID3 mRNA levels in HCEC cells after SMAD1, 5 or simultaneous SMAD 1 and 5 knockdown (KD) for 48 h followed by vehicle control or BMP4 stimulation for 24 h. All values are mean ± SEM. [file PATH-242-178-s002.tif]

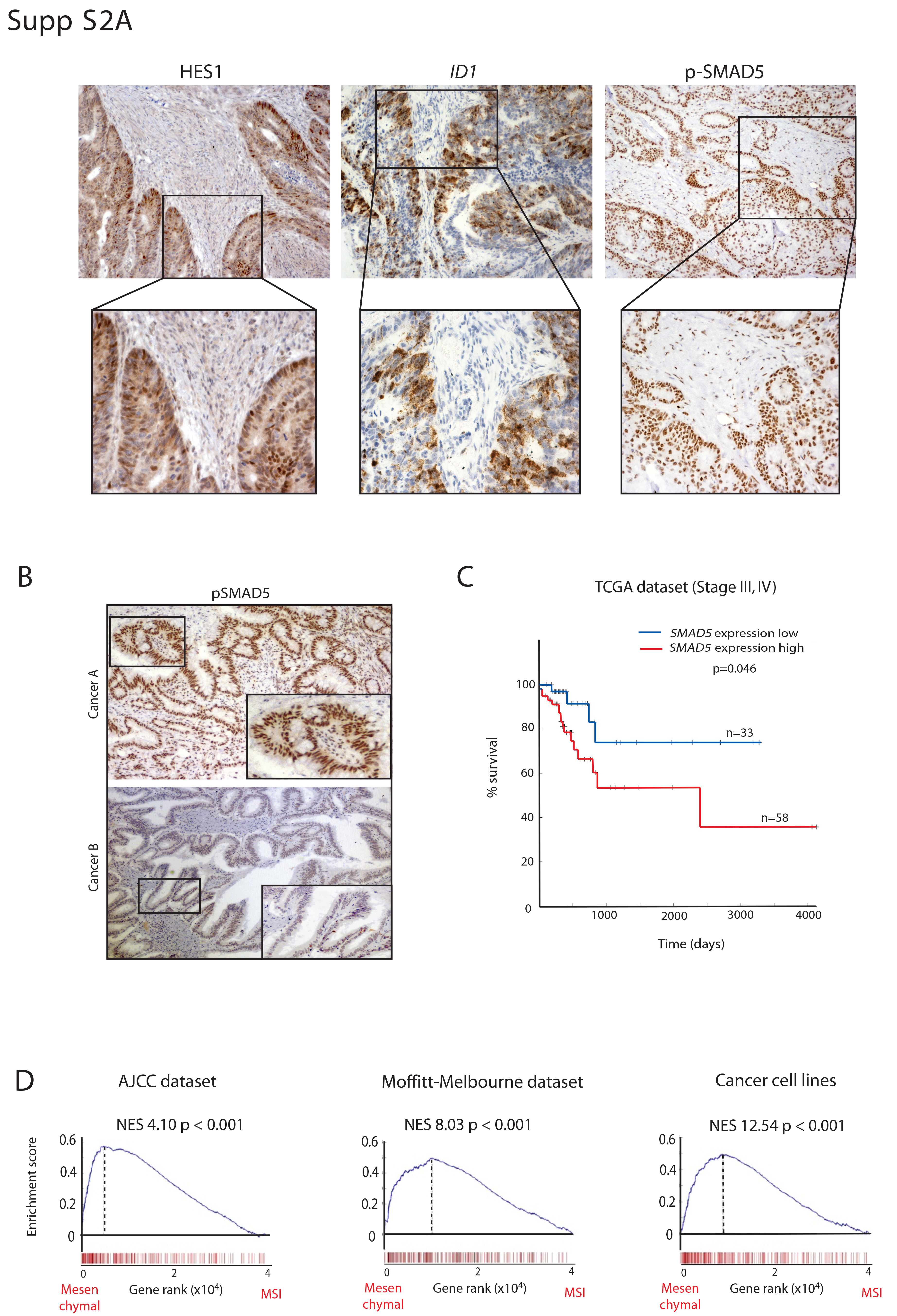

Supplement: Supplementary file 4 — Figure S2. Variable BMP and Notch signalling in different colorectal cancer molecular subtypes. 2A) Representative images of HES1, ID1 mRNA and p‐SMAD5 staining in human colorectal cancer samples showing staining predominantly restricted to the epithelial compartment with little or no stromal staining. 2B) Representative images of p‐SMAD5 staining in human colon tumours from a tissue microarray (n = 105) 2C) Kaplan‐Meier plot displaying recurrence‐free survival (RFS) over time in stage III and IV patients from the TCGA cohort. Log rank test p‐value compares RFS over time for patients grouped by KNN clustering according to SMAD5 expression levels. 2D) Gene set enrichment plots using 281 BMP signalling signature on two independent CRC datasets and a panel of colorectal cancer cell lines comparing mesenchymal versus MSI subtypes. P‐values generated by Kolmogorov–Smirnov statistics. [file PATH-242-178-s003.tif]

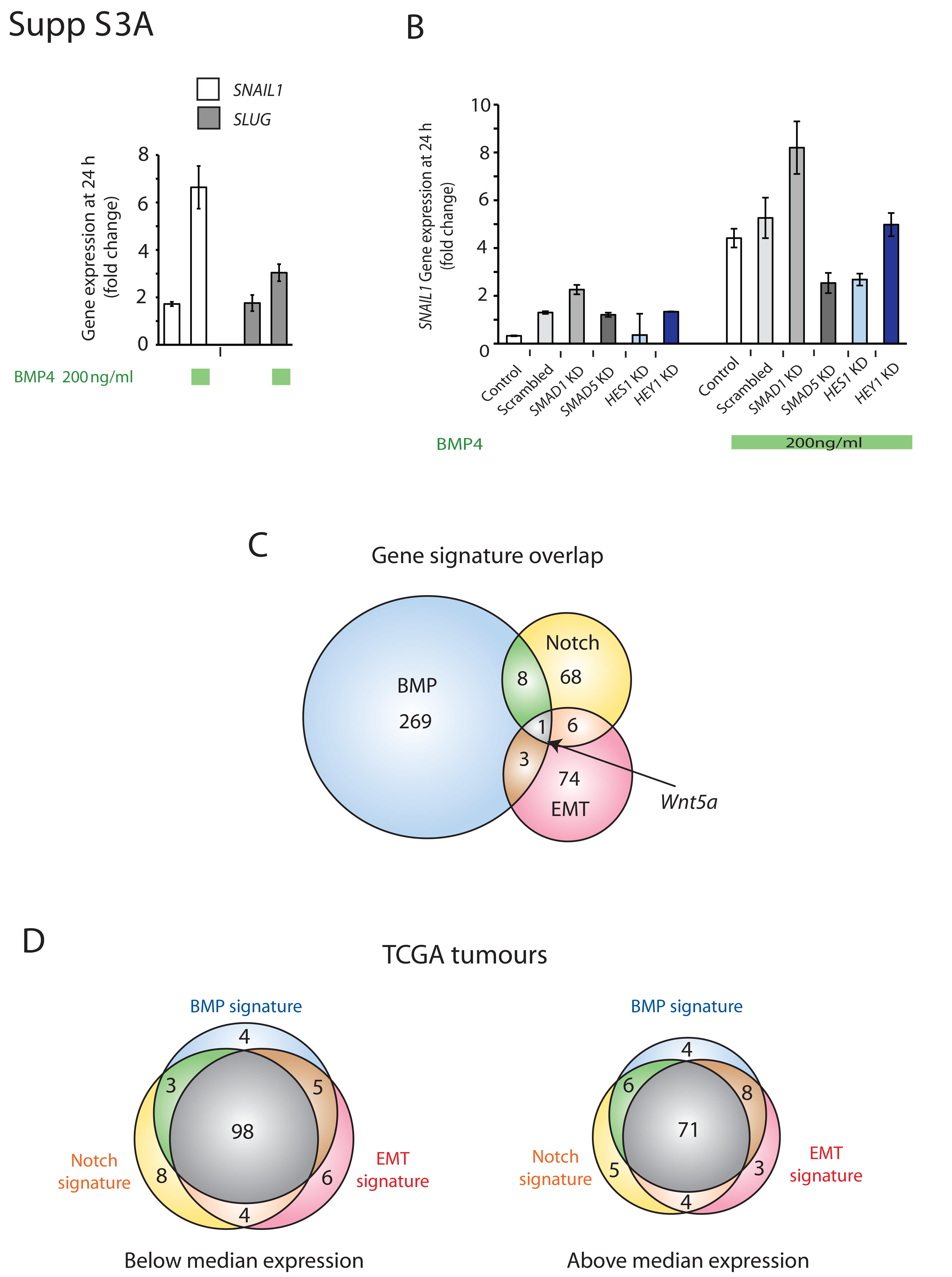

Supplement: Supplementary file 5 — Figure S3. Relative mRNA expression levels of SNAIL1 and SLUG in HCEC cells and overlap of BMP, Notch and EMT signatures in primary human colorectal cancers. 3A) Relative SNAI1 and SLUG mRNA levels in HCEC cells, 24 h after BMP4 treatment compared to control cells. Values are mean ± SEM (n = 2). 3B) Relative SNAI1 expression levels (fold change) in HCEC cells, 48 h after SMAD1, 5, HES1 or HEY1 knockdown (KD) and after 24 h of BMP4 treatment compared to control cells. Values are mean ± SEM (n = 2). 3C) Venn diagrams showing overlap of genes in generated BMP signature and curated Notch and EMT signatures. WNT5A is the only gene common to all signatures. 3D) Overlap of above and below median expression of BMP signalling, Notch and EMT gene signatures in numbers of different tumours in the TCGA dataset (Fishers exact test, p < 0.01). [file PATH-242-178-s004.tif]
