# Supplementary material for: Bone morphogenetic protein and Notch signalling crosstalk in poor‐prognosis, mesenchymal‐subtype colorectal cancer
Source: J Pathol. 2017 May 3;242(2):178–92. doi: 10.1002/path.4891 (PMC5488238; doi:10.1002/path.4891)
Supplement: Supplementary file 6 — Table S1. Primers used in chromatin immunoprecipitation experiments [file PATH-242-178-s005.doc]

**Table S1**. Primers used in chromatin immunoprecipitation experiments.

| **Primer name** | **Sequence** |
| --- | --- |
| HEY1 P1F | GGATCAGTGTGTGCGGAAC |
| HEY1 P1R | CACTCTCCTTCTCCACCTCG |
| HEY1 P2F | GAGGTGGAGAAGGAGAGTGC |
| HEY1 P2R | GCTGGAAGGCACCTAGGG |
| HEY1CONTF | GGCTCTGAATGGTTGGCAAA |
| HEY1 CONTR | AGAGCTGGGGTGGTGAATTC |
| HES1 P1R | TATATCTGGGACTGCACGCG |
| HES1 P1F | GGTCTAAGGCCCCAAATCCA |
| HES1 P2F | TGCCAGCTGATATAATGGAGAA |
| HES1 P2R | AGGGGCTGCAAAGAGATACA |
| HES1 CONTF | TCCAGAGATAATGCTTGCGC |
| HES1 CONTR | AGAATGTCCGCCTTCTCCAG |
| NOTCH1 PF | GGAAGAGAGGGCGGGAC |
| NOTCH1 PR | TGGGCGCCTACCTCGTG |
| NOTCH1 CONTF | CTGGAGCCGAGTTAGGAGG |
| NOTCH1 CONTR | CGGAGAAGCAACAGGAAACC |
| ID1 F | AGTCCGTCCGGGTTTTATG |
| ID1 R | TGTGTCAGCGTCTGAACCAG |
| GAPDH F | GAGCCTCGAGGAGAAGTTCC |
| GAPDH R | ACGACTGAGATGGGGAATTG |
